# The Effects of Pitch Manipulation on Male Ratings of Female Speakers and Their Voices

**DOI:** 10.3389/fpsyg.2022.911854

**Published:** 2022-07-07

**Authors:** Christina Krumpholz, Cliodhna Quigley, Karsan Ameen, Christoph Reuter, Leonida Fusani, Helmut Leder

**Affiliations:** ^1^Department of Cognition, Emotion, and Methods in Psychology, Faculty of Psychology, University of Vienna, Vienna, Austria; ^2^Konrad Lorenz Institute of Ethology, University of Veterinary Medicine, Vienna, Austria; ^3^Department of Behavioural and Cognitive Biology, University of Vienna, Vienna, Austria; ^4^Vienna Cognitive Science Hub, University of Vienna, Vienna, Austria; ^5^Department of Musicology, University of Vienna, Vienna, Austria

**Keywords:** cross-modal attractiveness, voice, sexual dimorphism, femininity, health, age, face, multisensory processing

## Abstract

Vocal and facial cues typically co-occur in natural settings, and multisensory processing of voice and face relies on their synchronous presentation. Psychological research has examined various facial and vocal cues to attractiveness as well as to judgements of sexual dimorphism, health, and age. However, few studies have investigated the interaction of vocal and facial cues in attractiveness judgments under naturalistic conditions using dynamic, ecologically valid stimuli. Here, we used short videos or audio tracks of females speaking full sentences and used a manipulation of voice pitch to investigate cross-modal interactions of voice pitch on facial attractiveness and related ratings. Male participants had to rate attractiveness, femininity, age, and health of synchronized audio-video recordings or voices only, with either original or modified voice pitch. We expected audio stimuli with increased voice pitch to be rated as more attractive, more feminine, healthier, and younger. If auditory judgements cross-modally influence judgements of facial attributes, we additionally expected the voice pitch manipulation to affect ratings of audiovisual stimulus material. We tested 106 male participants in a within-subject design in two sessions. Analyses revealed that voice recordings with increased voice pitch were perceived to be more feminine and younger, but not more attractive or healthier. When coupled with video recordings, increased pitch lowered perceived age of faces, but did not significantly influence perceived attractiveness, femininity, or health. Our results suggest that our manipulation of voice pitch has a measurable impact on judgements of femininity and age, but does not measurably influence vocal and facial attractiveness in naturalistic conditions.

## Introduction

Being judged to be attractive has been proposed to have positive effects on many aspects of our lives. Dion et al. (1972) described the “What is beautiful is good” stereotype, according to which physically attractive people are ascribed numerous positive characteristics. This was subsequently confirmed in empirical studies where attractive people were judged to be more social, intelligent, trustworthy, and healthy (Dion et al., 1972; Langlois et al., 2000; Rhodes et al., 2007; Coetzee et al., 2011; Ma et al., 2016). Moreover, attractive people have been found to be more successful in dating and short- and long-term relationships (Rhodes et al., 2005).

Attractiveness judgments are based on information acquired through different sensory modalities, and although both facial and vocal attractiveness have each been studied extensively (see Wells et al., 2009, for a review of multiple signals in humans), fewer studies have investigated attractiveness judgments of both modalities together. This is surprising, because research in other social domains has focused a lot on multisensory processing of audiovisual stimuli: In speech, the combination of facial and vocal speech has both facilitation and interference effects (see Campbell, 2007, for a review), with its prominent example, the McGurk effect, revealing completely different speech perception with differing sounds and accompanying lip movements (McGurk and MacDonald, 1976); In affective processing, the concurrent presentation of an affective voice changes emotion perception of facial expressions (Campanella and Belin, 2007); In identity processing, people are able to match speakers’ identities across video and auditory presentations (see Campanella and Belin, 2007, for a review of affective processing and identity processing). There is also evidence from other species that multisensory processing plays an important role in animal communication (Higham and Hebets, 2013) including mate choice. Several studies reported a benefit for senders or receivers when courtship was composed of multiple sensory modalities instead of single modalities for reasons such as improved signal efficiency, provision of information about multiple aspects of male quality, or generation of new information from the interaction of the different components (see Mitoyen et al., 2019 for an extensive overview). Multimodal signals can either be backup signals (Johnstone, 1996), offering the same information for higher accuracy, or multiple messages (Møller and Pomiankowski, 1993), offering unique or independent properties of an individual’s quality. Studies in humans have found evidence for redundant, but also non-redundant information from different sensory modalities (see Groyecka et al., 2017 for a review), and the relative importance of visual and auditory information for mate choice and their interaction is not yet disentangled.

Facial attractiveness is an important criterion in human mate choice and has been discussed as an indicator of reproductive success regarding direct benefits, whereby the perceiver directly gains for themselves or for their offspring, and indirect benefits, whereby the perceiver gains genetic benefits for their offspring (Little et al., 2011). Several features determining facial attractiveness have been studied across cultures, including facial symmetry (Scheib et al., 1999; Penton-Voak et al., 2001; Jones et al., 2004; Wells et al., 2009; Saxton et al., 2011; Zheng et al., 2021), averageness (Langlois and Roggman, 1990; Roberts et al., 2005; Vingilis-Jaremko and Maurer, 2013; Lee et al., 2016), and sexual dimorphism (Alley and Cunningham, 1991; Langlois et al., 2000; Rhodes, 2006; Mogilski and Welling, 2017; Hu et al., 2018; Fiala et al., 2021). Regarding the latter, evolutionary explanations hypothesize that traits which emphasize femininity or masculinity of a face are cues to underlying aspects of mate quality such as fertility, fecundity, or general health and therefore contribute to attractiveness judgements (Gangestad and Scheyd, 2005). For men, the immunocompetence hypothesis suggests that facial masculinity is a handicap signal, similarly to other androgen-dependent traits: testosterone has several costs which only the healthiest men who appear to have the best condition can afford (Folstad and Karter, 1992; Chen and Parker, 2004). For women, femininity in faces is considered as a relevant cue to fertility due to its relationship with estrogen (Smith et al., 2006), and women with feminine faces have been found to be healthier (Gray and Boothroyd, 2012). However, other studies showed contradictory results, where facial femininity neither showed a relationship with actual health (Jones, 2018) nor with immune function (Cai et al., 2019), and women with higher facial attractiveness did not show higher levels of estradiol (Jones et al., 2018).

Voice attractiveness also seems to be important in human mate choice and is a good indicator of female fertility on different time scales, because it peaks during a woman’s most fertile years (Röder et al., 2013) and during the late follicular phase of the ovulatory cycle (Pipitone and Gallup, 2008) due to fluctuations in sex hormones (Abitbol et al., 1999; Amir and Biron-Shental, 2004). Apart from voice averageness (Winkielman et al., 2006; Bruckert et al., 2010), voice pitch has been discussed as an important and stable acoustic-phonetic parameter related to femininity and voice attractiveness (Pisanski et al., 2018). Voice pitch is closely related to the fundamental frequency (f_0_), whereby f_0_ describes the actual physical phenomenon and voice pitch our perception of f_0_, i.e., how we interpret the signal. Accordingly, several studies found that men judge female voices with higher f_0_ as more attractive than female voices with lower f_0_ (Apicella and Feinberg, 2009; Little et al., 2011; Pisanski et al., 2018; Mook and Mitchel, 2019), and this effect was even found when f_0_ was higher than their average female f_0_ of 200 Hz (Collins and Missing, 2003; Feinberg et al., 2008). Similarly, voices that were increased in f_0_, i.e., feminized, were always preferred over voices that were lowered in f_0_, i.e., masculinized (Puts et al., 2011). On average, male f_0_ is about half that of female f_0_ (Dabbs and Mallinger, 1999), which makes f_0_ and voice pitch good indicators of sexual dimorphism.

As this wide range of literature shows, there is evidence for attractiveness cues in both voices and faces, and both facial and vocal attractiveness were related to correlates of reproductive capability such as testosterone levels, age, and body mass index (Wheatley et al., 2014). Nonetheless, it remains unclear to what extent both modalities interact. Some studies have investigated the correlation between facial and vocal attractiveness and found that women who received high attractiveness ratings for images of their faces also received high attractiveness ratings for recordings of their voices, indicating that vocal and facial attractiveness are related and naturally co-occur (Zuckerman et al., 1991; Collins and Missing, 2003). A recent experimental study of cross-modal effects (Mook and Mitchel, 2019) investigated their possible mutual influence by manipulating f_0_ and face averageness, and asking male raters to judge female vocal or facial attractiveness of unimodal and audiovisual stimuli. They used 6 manipulation levels for f_0_ (in 20 Hz increments from 160 Hz to 260 Hz) and facial averageness (averages created from 1, 2, 4, 8, 16, or 32 faces). In their unimodal conditions, they found that increased f_0_ and facial averageness led to increased vocal and facial attractiveness, respectively. Vocal attractiveness was most affected when f_0_ was increased from 160 to 180 Hz and least affected by increases from 200 to 220 Hz. In audiovisual conditions, participants were instructed to ignore one modality (which was manipulated) and rate the other. When faces were to be ignored and voices rated, variations in facial averageness nevertheless led to changes in ratings of vocal attractiveness, providing evidence for a cross-modal influence of facial attractiveness on judgements of voices. On the other hand, when voices were to be ignored, variations in f_0_ did not lead to changes in ratings of facial attractiveness, suggesting that the cross-modal influence is not symmetric for attractiveness ratings.

These results provide interesting evidence for a cross-modal interaction of vocal and facial attractiveness, but it remains unclear whether they will generalize to more naturalistic settings due to several reasons: First, and most important, the authors used combinations of static images of faces and voice recordings, which decreases the possibility that participants integrate voice and face because static images lack any changes over time. In contrast, temporally simultaneous properties of dynamic stimuli (e.g., real-life situations or videos) promote integration by offering information about intermodal properties such as lip movements (Sumby and Pollack, 1954), rate (Munhall et al., 1996), or rhythm (Bahrick and Lickliter, 2004), and these simultaneous properties are drastically reduced or non-existent when static images are combined with voice recordings (Lander, 2008). Moreover, vowels were used as audio stimuli (as well as in Collins and Missing, 2003). Prior studies have found that vowels might not deliver relevant cues of fertility (Lindholm et al., 1997; Bryant and Haselton, 2009; Fischer et al., 2011), and it has been suggested that judgments of voice traits can differ between short speech sounds (e.g., vowels) and longer trains of speech as in real-life encounters (Pisanski and Feinberg, 2018). Sentences in particular seem to convey important voice characteristics such as phonetic or prosodic differences between men and women (Simpson, 2009), and hence, might also be relevant for personal judgments such as attractiveness or femininity (see also Zäske et al., 2020).

### Research Aims and Hypotheses

In this study, we asked two questions: First, does voice pitch influence voice judgments of attractiveness, femininity, health, and age in more naturalistic conditions? Second, does voice pitch have a cross-modal effect on judgments of facial attractiveness, femininity, and health under more naturalistic, dynamic conditions?

Our approach differs from that of previous studies in that we use speech stimuli, which represent real-life encounters more accurately than non-speech vocalizations like vowel sounds. Most previous literature examining the effects of voice pitch on vocal judgments (e.g., Collins and Missing, 2003; Mook and Mitchel, 2019) used non-speech vocalizations. Although some argue that the use of these short, neutral vowel sounds is sufficient or even beneficial, because voice characteristics can be conveyed without contextual factors such as co-articulation, emphasis, and semantic meaning (Ferdenzi et al., 2013), and it facilitates analysis of acoustic measures (Patel et al., 2011), the raters’ evaluations are clearly different to a real-life situation (see Ferdenzi et al., 2013, for differences in perceived attractiveness of vowel and word stimuli). We hypothesize that if voice pitch is used in judgments of attractiveness, femininity, health, and age of voices under the more naturalistic conditions of verbal speech stimuli, its manipulation should result in changed ratings.

Our approach also differs from previous studies in that we use dynamic visual stimulus material. Most studies used a combination of static images and voice recordings. This approach certainly allows for control over stimuli and more possibilities to manipulate and combine different voices and faces, but it ignores temporally synchronous properties of speech. In real-life encounters we seldom rely on a single modality to judge a person’s traits, and these modalities are seldom presented in a combination of static images and voice recordings, but mostly in a synchronized way, e.g., we can see someone’s lips and eyebrows moving while they are speaking. Previous studies found significant differences in the correlation of visual and vocal attractiveness and general attractiveness judgments between static and dynamic faces (Lander, 2008), and suggest different evaluative standards underlying static and dynamic presentations (Rubenstein, 2005). Therefore, it is relevant to study voice and face in a more naturalistic setting by presenting both modalities in the form of synchronized videos. We hypothesize that if voice pitch has a cross-modal effect on face perception, it should influence perceived attractiveness, femininity, health, and age of faces in synchronized video material.

Previous studies on the effects of sexual dimorphism on attractiveness revealed sex differences, with effects found to be smaller (e.g., Rhodes, 2006) and more ambiguous for women’s ratings of male faces (for a review see Little et al., 2011). For these reasons, we restricted the present study to hetero- or bisexual men’s assessments of female faces.

## Materials and Methods

### Participants

Across two waves of data collection, a total of *N* = 104 hetero- or bisexual men with a mean age of 24.30 years (SD = 4.51, Range: 19–38) completed our study. Psychology student participants (*N* = 90, *M* = 24.02 years, Range: 19–38) took part to gain course credit, whereas non-psychology student participants (*N* = 14, *M* = 26.23 years, Range: 20–37) received a monetary compensation (15€). All participants reported normal or corrected-to-normal vision and hearing. Prior to the experiment, participants were thoroughly instructed and gave their informed consent in written form, with the knowledge that they could withdraw at any time from the experiment without any further consequences. Afterward, all participants were given verbal and written information about the theoretical background, study design, and hypotheses.

### Ethics Statement

This study was conducted in accordance with the Declaration of Helsinki (revised, 1983) and was ethically approved by the Ethics Committee of the University of Vienna (title of project “Comparative aesthetics,” reference number 00376).

### Materials

**Figure 1:**
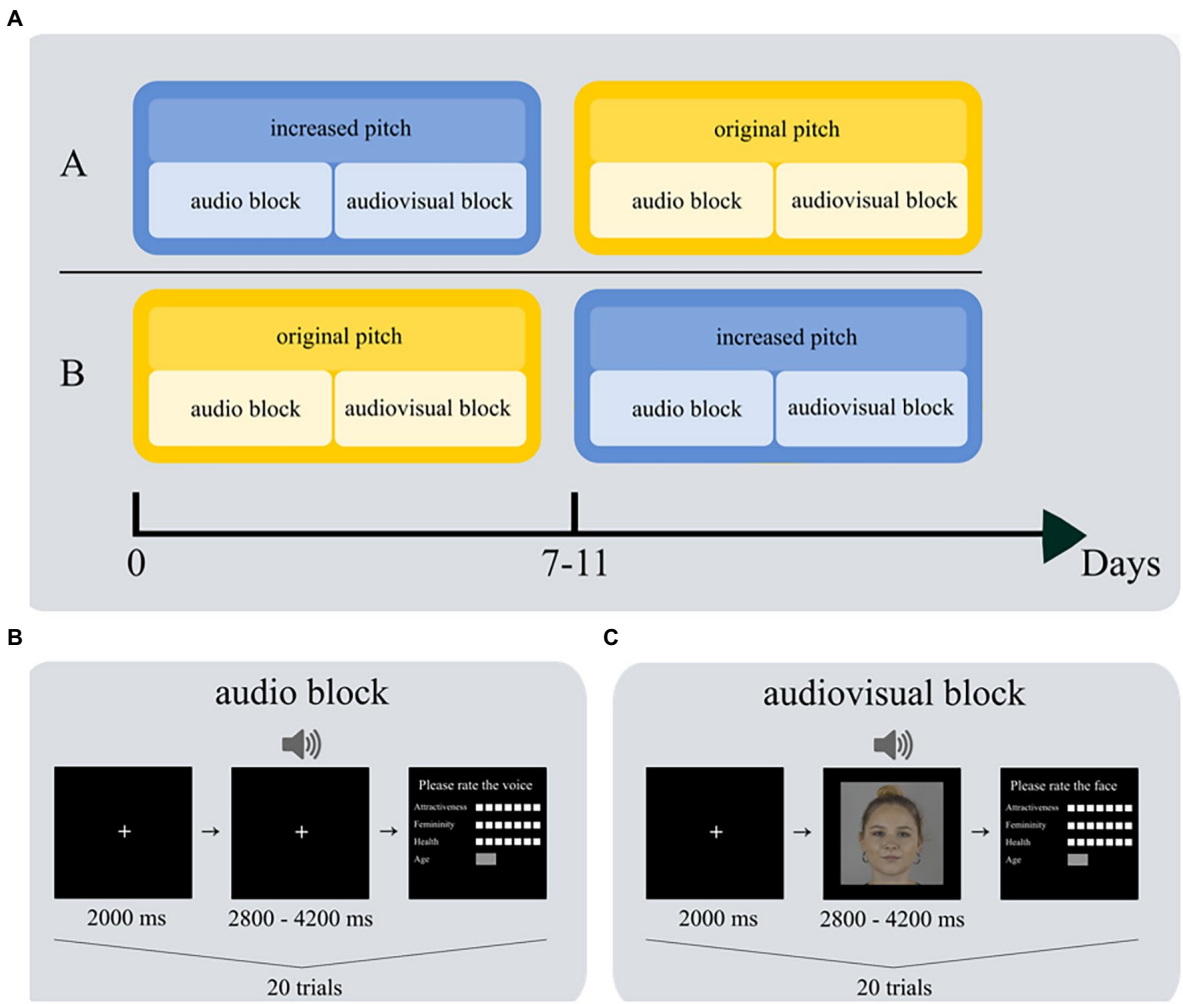
Experimental set-up. **(A)** Participants were randomly assigned to order A or B. They completed two sessions with 7–11 days in between. On each session, they first completed the audio block and then the audiovisual block; sessions differed regarding voice pitch. Each participant completed 20 trials in the audio block **(B)** and 20 trials in the audiovisual block **(C)**. The depicted face is for example purposes only; although it is not contained in the database, it was recorded under the same conditions.

The experiment consisted of two blocks, audio and audiovisual, which both used stimulus material from the neutral emotional expression category of the Vienna Talking Faces database (ViTaFa; Krumpholz et al., unpublished). Recordings of 20 women between the ages of 18 and 45 containing two different spoken sentences without any deeper meaning were chosen: *Morgens ist auf den Straßen viel los* (The streets are busy in the morning) and *Die Leute sitzen vor der Tür* (People sit outside the door). In the audiovisual block, we used synchronized videos, and in the audio block, we used the audio track from the same videos. To avoid that participants could recognize stimuli from the audio block in the audiovisual block, we block-randomized the sentences over blocks and participants. One voice pitch condition (original or increased) was presented in each block (see Figure 1A).

#### Audiovisual Block

In the *audiovisual block*, we presented synchronized video recordings. Videos were originally recorded at a frame rate of 30 frames per second and resolution was downscaled to 800×800 pixels for this experiment (approximately 18 degrees of visual angle in width and height). Each face was centrally aligned on a gray background, so that the head accounted for exactly 80% of the height and the nasion was on the vertical centerline of the screen. Light conditions were kept identical for all video recordings. All voices were recorded at 48 kHz sampling rate and 16-bit dynamic range and were equalized regarding their volume to avoid differences in perceived loudness between stimuli (Scherer et al., 1973).

#### Audio Block

In the *audio block*, only the audio track was presented. The audio track differed from the audiovisual block content-wise: Participants heard a different sentence in both blocks. During the presentation of the audio track, a white fixation cross remained in the middle of the screen on a black background to direct participants visual attention to the screen as a better comparison to the audiovisual block. However, we did not instruct participants to fixate the cross to make it comparable to the audiovisual block, where there was also no fixation instruction.

#### Voice Pitch Manipulation

Following Vukovic et al. (2010b), f_0_ was calculated for every original voice using Praat’s (Boersma and Weenink, 2007) autocorrelation function (Boersma, 1993) with input parameters set at 100 Hz for pitch floor, 600 Hz for pitch ceiling, and 0.0075 s as measurement interval. The resulting f_0_ of the original voices ranged from 171 Hz to 267 Hz (see Supplementary Table 1 for an overview of voice pitch of all stimuli). For the voice pitch manipulation, we shifted f_0_ of each voice recording using the pitch-synchronous overlap-add (PSOLA) algorithm (Boersma and Weenink, 2007) in PRAAT by 0.5 equivalent rectangular bandwidths (ERBs) to create a resynthesized vocal stimulus whose f_0_ was increased by approximately 20 Hz. Examples of voice recordings are available on request. Other voice parameters such as breathiness, formant dispersion, or articulation and nasality (Zuckerman and Miyake, 1993) were allowed to vary naturally, so that pitch was the only difference between conditions.

#### Apparatus

The experiment was programmed with OpenSesame (version 3.2.8) and conducted on a desktop computer with Windows 10 Enterprise. Visual stimuli were presented on a gray background on a 24” LCD-screen (LG 24MB65PM; native resolution 1920 × 1,200 pixels) with a frame rate of 60 Hz. Audio settings were identical for all participants and sounds were presented *via* headphones (Sony MDR 7506 or Sennheiser HD 380 Pro). Participants were seated in front of the monitors without a chin rest with an approximate distance to the screen of 65 cm. They provided ratings *via* keypress or mousDe click. Responses were not timed and participants were instructed to give spontaneous ratings.

### Procedure

The experiment was conducted in a testing room at the faculty of Psychology of the University of Vienna. In a within-subject design, participants were invited to the laboratory twice. In the first session, they were randomly assigned to either the original pitch or the increased pitch condition. To decrease the probability that participants remembered their ratings from the first session, the second session took place after a break of 7–11 days depending on the participants’ availability. This approach is quite common in our research laboratory (e.g., see Leder et al., 2016, for a similar interval; Specker et al., 2020, and Fekete et al., 2022, for even shorter intervals), but of course it does not guarantee that participants will not remember any of their ratings. The procedure was nearly the same in both sessions: Prior to the experiment, participants gave informed consent and were provided with the instructions. They were informed that voice and video recordings would be presented and that their task was to rate perceived attractiveness, femininity, health, and age of each voice and each face afterward. In the first session, they additionally completed a demographics questionnaire. Then, the actual experiment started, and participants completed the audio block first, followed by the audiovisual block (see Figure 1A for an illustration of the experimental design). We chose this order so that participants in the audio block would be naïve, i.e., they would not be able to recognize voices from the videos and thus the evaluation of the voices would not be biased by prior experience. We remind the reader that the sentence material was different in each block to minimize recognition effects in the audiovisual block.

At the beginning of each block, a practice trial congruent to the actual task was performed, so that participants became familiar with the task, which differed between blocks. Each trial began with the presentation of a white fixation point, centered on a black background, that lasted for 2000 ms. In the audio block (Figure 1B), the fixation point remained on the screen, and a voice recording was played simultaneously; each voice recording lasted between 2.8 and 4.2 s. In the video block (Figure 1C), the fixation cross was replaced with a video at the same position, also lasting between 2.8 and 4.2 s. The end of a trial was marked by a response screen where participants had to provide ratings of perceived attractiveness, femininity, and health on a 7-point Likert scale, and had to estimate age in years in a free answer format. The target of the rating differed depending on the block: participants were instructed to give ratings for the *voice* in the audio block and ratings for the *face* in the audiovisual block. Participants completed 20 trials in the audio block and 20 trials in the audiovisual block.

To validate that the difference in voice pitch was consciously perceptible despite the relatively small manipulation of about 20 Hz, we included a pitch discrimination task after the experiment in session 2: In a two-interval forced choice (2-IFC) format, we presented five pairs of the unmanipulated and the manipulated version of the same sentence and speaker of randomly chosen voice recordings from the experiment, and participants were instructed to indicate which interval had the higher pitch. Each pair was included twice, using both possible presentation orders, i.e., manipulated first or unmanipulated first, resulting in 10 trials. After the pitch discrimination task, we asked participants whether they noticed anything unusual in the experiment and informed them about the study objectives.

### Statistical Analyses

All data and analysis scripts are available at the Open Science Framework.^1^ All statistical analyses were conducted in RStudio (version 1.4.1717). We excluded three participants’ ratings from the analysis of femininity ratings in both blocks, because they indicated in the debriefing that they rated all voices and faces as equally feminine.

We employed Linear Mixed Models (LMM) to investigate the influence of voice pitch on ratings of attractiveness, femininity, health, and age, and we included random slopes to account for (a) individual differences, because there is growing evidence that private taste accounts for as much variance in ratings as shared taste (Tanaka et al., 2020) and (b) for differences between stimuli, because increasing f_0_ by 20 Hz influences voice pitch differently relative to f_0_, and because we let other voice parameters apart from voice pitch such as breathiness or formant dispersion vary naturally and hence, voice pitch could influence each voice differently. We used the lme4 package (Bates et al., 2015) to perform four linear effects analyses of the relationship between voice pitch (voicePitch; manipulated variable; original vs. increased) and ratings from the audio block of attractiveness, femininity, health, and age of voices (ratingResponse; dependent variables). We also performed four linear effects analyses of the relationship between voice pitch (voicePitch; manipulated variable) and ratings from the audiovisual block of attractiveness, femininity, health, and age of faces (ratingResponse; dependent variables). All 7-point Likert scales were treated as quasi-metric. As fixed effect, we entered voice pitch into the models. As random effects, we included intercepts for subjects (subjectNr) and stimuli (stimulusNr), as well as by-subject and by-stimulus random slopes for the effect of voice pitch to account for different effects per person and per stimulus. The full model specification was as follows:


$$\begin{array}{l} \mathrm{ratingResponse}\sim \mathrm{voicePitch}+\left(1+\mathrm{voicePitch|subjectNr}\right)+\\ \;\;\;\;\;\;\;\;\;\;\;\;\;\;\;\;\;\;\;\;\;\;\;\;\;\;\;\;\;\;\;\;\;\;\;\;\;\;\;\;\;\;\;\;\;\;\;\;\;\;\;\;\;\;\;\;\;\;\;\;\;\;\;\;\;\;\;\;\;\;\;\;\;\;\;\;\;\;\left(1+\mathrm{voicePitch|stimulusNr}\right) \end{array}$$

However, for some ratings we had to simplify the models, because they failed to converge or showed a boundary fit due to overfitting. We then first removed the random effect of the stimulus and if needed, the random slope from the subject number. An overview of which model was used for which rating is illustrated in Supplementary Table 2. For the sake of completeness, we calculated all simplified model versions also for those ratings where the more complex models converged. The interested reader can find all of these analyses in our repository at the Open Science Framework (see Footnote 1).

Visual inspection of residual plots against fitted values did not reveal any obvious deviations from homoscedasticity. Residuals were non-normally distributed; however, a deviation from normality seems to be less crucial than heteroscedasticity because it becomes more likely with larger sample sizes, and LMMs seem to be robust against non-normality when outliers are dealt with (Knief and Forstmeier, 2021). To assess the validity of the mixed effect analyses, we performed likelihood ratio tests comparing the full models with the effect in question against the null model with only the random effects structure. The null model specification was as follows:


$$\begin{array}{l} \mathrm{ratingResponse}\sim \left(1+\mathrm{voicePitch|subjectNr}\right)+\\ \;\;\;\;\;\;\;\;\;\;\;\;\;\;\;\;\;\;\;\;\;\;\;\;\;\;\;\;\;\;\;\;\;\;\;\;\;\;\;\;\;\;\;\;\;\left(1+\mathrm{voicePitch|stimulusNr}\right) \end{array}$$

Wherever convergence or overfitting of the full model specification had to be altered, we also adapted the null models by removing the random effect of stimulus number and the random slope of subject number, i.e., the random effects structure was identical within a given model comparison. We rejected results in which the model with the effect in question did not differ significantly from the null model (see Table 1 for an overview of the model comparisons). Throughout the paper, we present *p*-values that are considered significant at the level of *α* = 0.05.

**Table 1:** Model comparisons of the full linear models with the effect (voice pitch) in question against the null linear models without the effect in question.

Response variable	Rating	*χ* ^2^	*p*
Attractiveness	Voices	2.331	0.127
	Faces	0.594	0.441
Femininity	Voices	9.856	0.002^**^
	Faces	0.648	0.421
Health	Voices	1.218	0.270
	Faces	0.150	0.699
Age	Voices	31.526	< 0.001^***^
	Faces	7.358	0.007^**^

Ratings for attractiveness, femininity, and health were given on a 7-point scale. Age ratings were given via free input field.

** *p* < 0.01 and

*** *p* < 0.001.

## Results

### Pitch Discrimination Task

**Figure 2:**
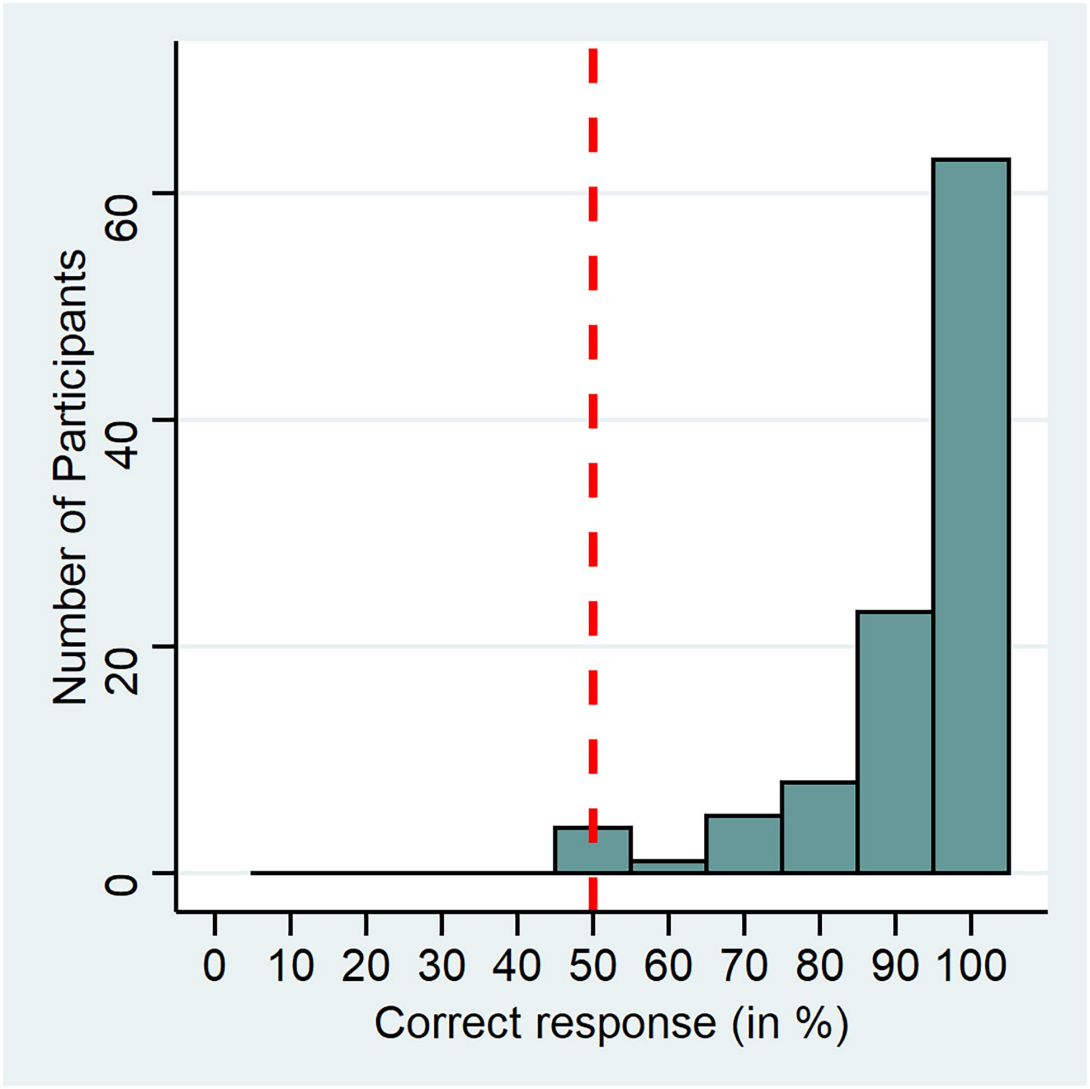
Pitch discrimination task performance. The x-axis shows the percentage of correct responses from the 10 trial 2-IFC experiment, the y-axis shows the number of participants for each bin. The dashed line shows chance level (50%).

Results of the pitch discrimination task are visualized in Figure 2. We calculated a one-sample sign test (to handle a non-symmetric distribution) to compare group performance with the chance level of 50 percent. It revealed a significant result (*S* = 100, *p* < 0.001), which indicates that participants were on average able to discriminate between original and increased voice pitch. In the debriefing, no participant indicated that they were aware of the pitch manipulation or mentioned any perceived artificiality of the voice in the manipulated condition.

### Descriptive Statistics

**Figure 3:**
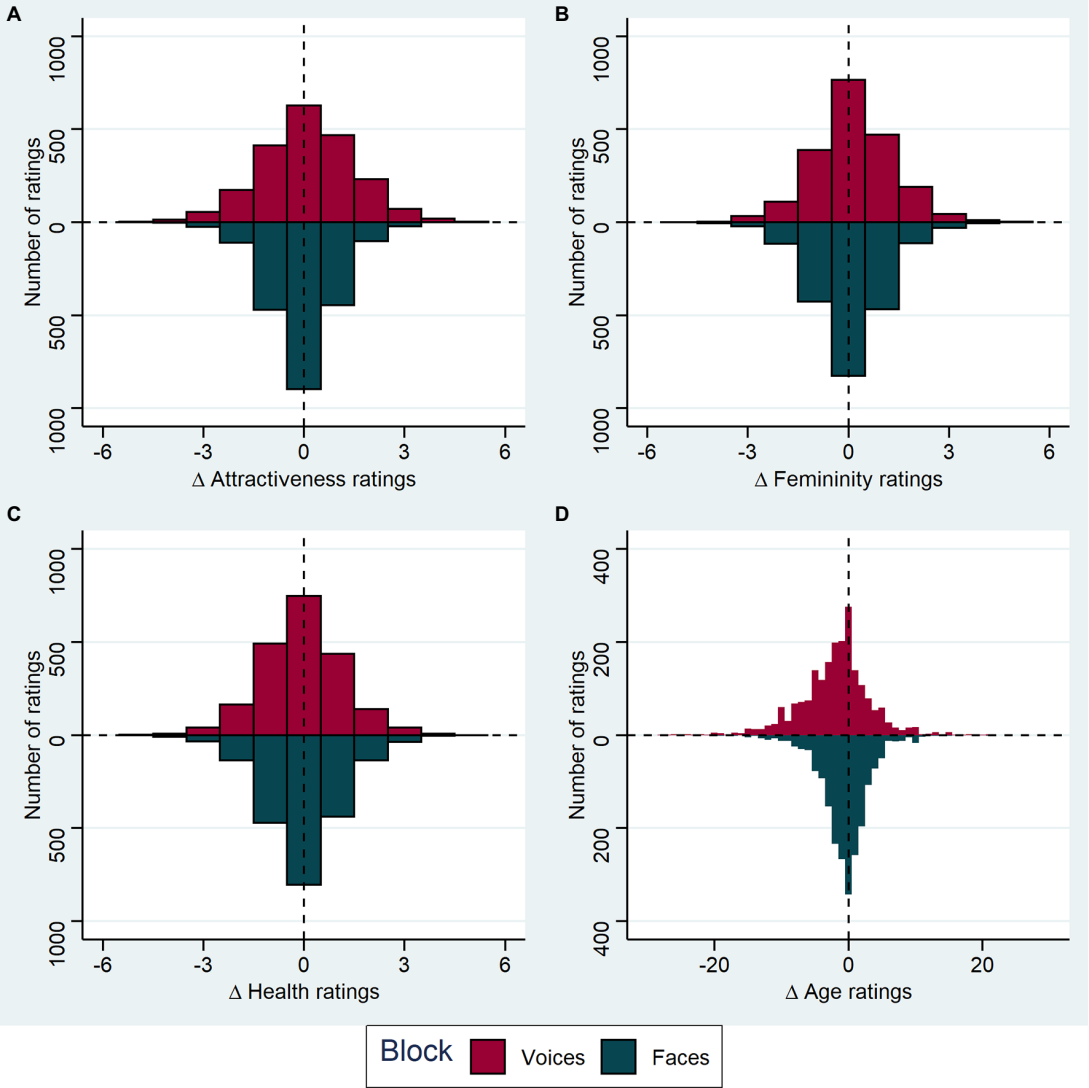
Effect of pitch manipulation on ratings. **(A)** The distribution of attractiveness rating differences for increased pitch minus original pitch is shown as a histogram, with results from the audio block (voice ratings) shown above the horizontal line (dark pink bars) and results from the audiovisual block (face ratings) shown below (dark cyan bars). Each stimulus and subject combination is included (for voices *n* = 2078, for faces *n* = 2075); ratings were given on a 7-point Likert scale. **(B)** as in **(A)**, for femininity ratings (for voices *n* = 2016, for faces *n* = 2012). **(C)** Health ratings (for voices *n* = 2076, for faces *n* = 2076). **(D)** Age ratings (for voices *n* = 2059, for faces *n* = 2068). Age was rated in years.

Means and standard deviations for all judgments of voices in voice recordings can be found in Table 2, for judgments of faces in video recordings in Table 3. We also visualized the rating differences between original and increased voice pitch for each stimulus rated by each participant in both blocks in Figure 3.

**Table 2:** Means and standard deviations of voice ratings with original and increased pitch.

Variable	Original pitch		Increased pitch	
	*M*	*SD*	*M*	*SD*
Attractiveness	3.89	0.68	4.00	0.73
Femininity	5.00	0.65	5.16	0.67
Health	5.03	0.71	4.98	0.69
Age	27.20	3.11	25.20	2.74

Ratings for attractiveness, femininity, and health were given on a 7-point scale. Age ratings were given via free input field.

**Table 3:** Means and standard deviations of face ratings with original and increased pitch.

Variable	Original pitch		Increased pitch	
	*M*	*SD*	*M*	*SD*
Attractiveness	3.50	0.72	3.47	0.72
Femininity	4.54	0.75	4.58	0.75
Health	4.43	0.75	4.41	0.71
Age	27.20	2.29	26.70	2.31

Ratings for attractiveness, femininity, and health were given on a 7-point scale. Age ratings were given via free input field.

### Audio Block

**Figure 4:**
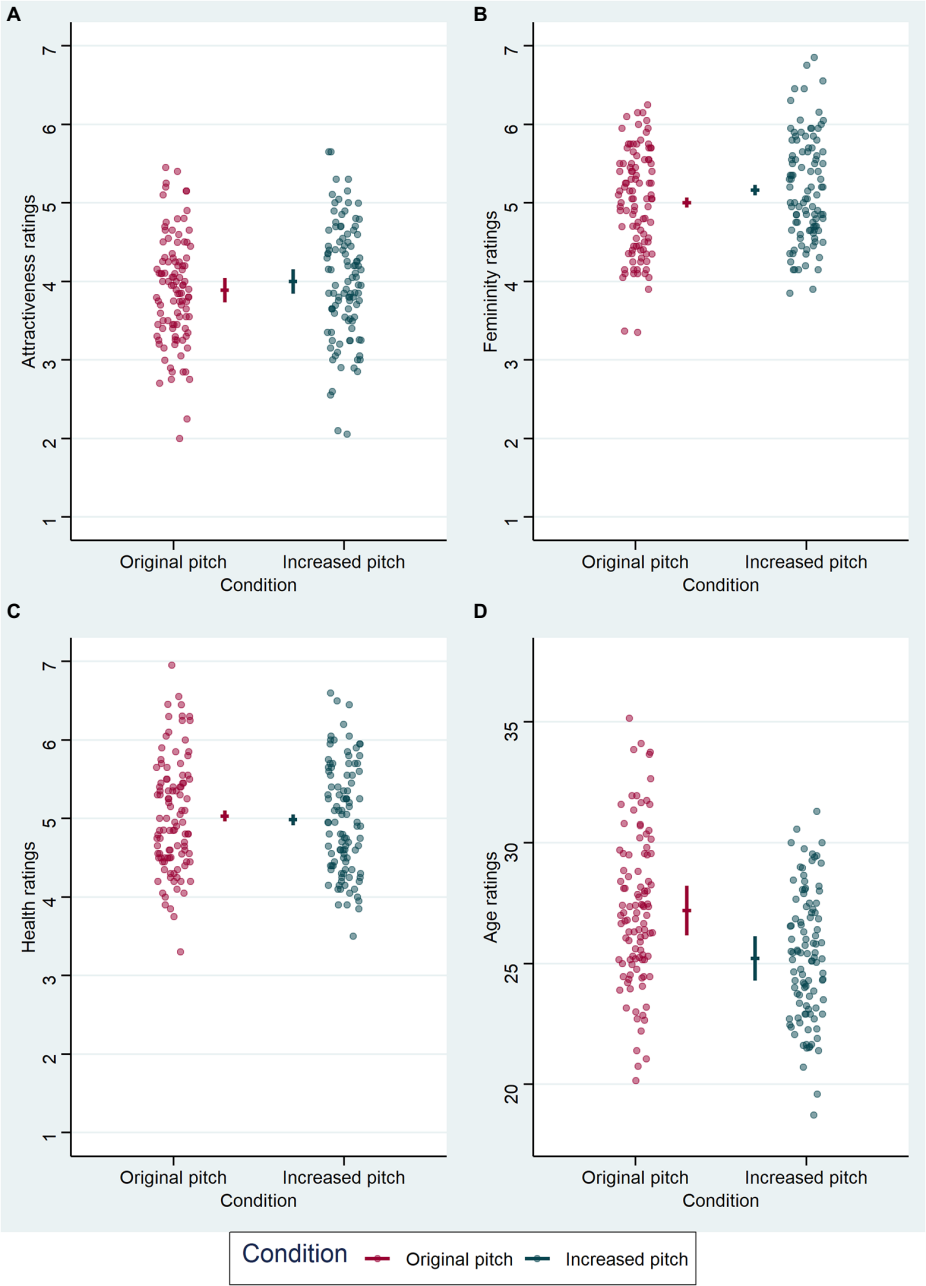
Results from the LMM analyses of voice ratings in the audio block. Each graph represents the effect of voice pitch on the respective voice judgment: **(A)** for attractiveness ratings, **(B)** for femininity ratings, **(C)** for health ratings, and **(D)** for age ratings (in years). Condition is indicated on the x-axis and color-coded, original voice pitch in dark pink and increased voice pitch in dark cyan. Data points (circles) represent mean values per subject and are jittered horizontally for visualization purposes. Horizontal lines show model estimate for each condition and error bars represent 95% confidence intervals of the model fit.

Results from the audio block are visualized in Figure 4, model comparisons are displayed in Table 1. LMM analyses (Table 4) revealed a significant effect of voice pitch on perceived femininity (*β* = 0.159, *t* = 3.218, *p* = 0.002) in voice recordings, with increased pitch eliciting higher ratings of femininity of voices than original pitch. There was also a significant effect of voice pitch on perceived age (*β* = −1.982, *t* = −7.931, *p* < 0.001) in voice recordings, with increased pitch leading to lower age ratings than original pitch. The effects of voice pitch on perceived attractiveness (*β* = 0.113, *t* = 1.543, *p* = 0.128) and health (*β* = −0.048, *t* = −1.107, *p* = 0.271) in voice recordings were non-significant.

**Table 4:** Linear mixed models with voice pitch condition (original vs. increased) as the fixed effect and ratings of attractiveness, femininity, health, and age of voices as the dependent variable.

Predicted variable	Predictor	Estimate	SE	Test (*df*)	*p*
Attractiveness	Intercept	3.886	0.156	25.013 (27.662)	
	Voice pitch	0.113	0.073	1.543 (56.533)	0.128
Femininity	Intercept	5.002	0.064	77.858 (100.926)	
	Voice pitch	0.159	0.050	3.218 (101.032)	0.002^**^
Health	Intercept	5.031	0.069	72.492 (104.023)	
	Voice pitch	−0.048	0.044	−1.107 (104.025)	0.271
Age (years)	Intercept	27.192	1.024	26.556 (23.547)	
	Voice pitch	−1.982	0.250	−7.931 (30.665)	<0.001^***^

SE, standard error; df, degrees of freedom.

** *p* < 0.01

and *** *p* < 0.001.

### Audiovisual Block

**Figure 5:**
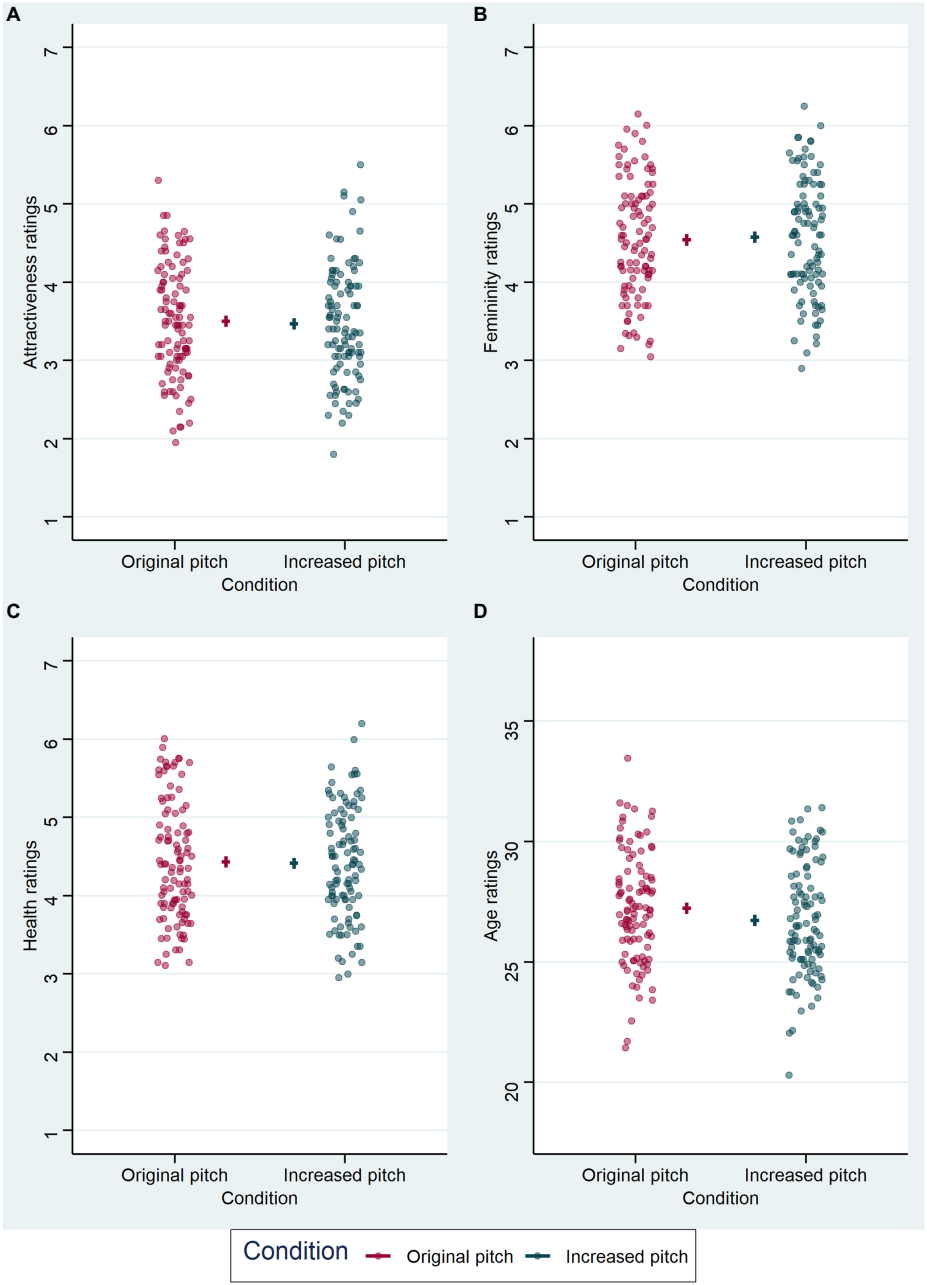
Results from the LMM analyses of face ratings in the audiovisual block. Each graph represents the effect of voice pitch on the respective face judgment: **(A)** for attractiveness ratings, **(B)** for femininity ratings, **(C)** for health ratings, and **(D)** for age ratings (in years). Condition is indicated on the x-axis and color-coded, original voice pitch in dark pink and increased voice pitch in dark cyan. Data points (circles) represent mean values per subject and are jittered horizontally for visualization purposes. Horizontal lines show model estimate for each condition and error bars represent 95% confidence intervals of the model fit.

Results from the audiovisual block are visualized in Figure 5, model comparisons are displayed in Table 1. LMM analysis (Table 5) also revealed a significant effect of voice pitch on perceived age (*β* = −0.5, *t* = −2.714, *p* = 0.007) in faces, with increased pitch leading to lower age ratings than original pitch. However, the effect of voice pitch on perceived femininity (*β* = 0.035, *t* = 0.806, *p* = 0.422) was no longer significant for face ratings. Effects of voice pitch on perceived attractiveness (*β* = −0.033, *t* = −0.772, *p* = 0.442) and health (*β* = −0.019, *t* = −0.387, *p* = 0.699) of faces were non-significant.

**Table 5:** Linear mixed models with voice pitch condition (original vs. increased) as the fixed effect and ratings of attractiveness, femininity, health, and age of faces as the dependent variable.

Predicted variable	Predictor	Estimate	SE	Test (*df*)	p
Attractiveness	Intercept	3.500	0.070	49.918 (104)	
	Voice pitch	−0.033	0.043	−0.772 (103.617)	0.442
Femininity	Intercept	4.540	0.074	61.342 (100.988)	
	Voice pitch	0.035	0.044	0.806 (101.161)	0.422
Health	Intercept	4.431	0.073	60.320 (103.996)	
	Voice pitch	−0.019	0.049	−0.387 (103.991)	0.699
Age (years)	Intercept	27.222	0.229	118.703 (147.741)	
	Voice pitch	−0.500	0.184	−2.714 (4044.088)	0.007^**^

SE, standard error; df, degrees of freedom.

** *p* < 0.01.

### Exploratory Analyses

**Figure 6:**
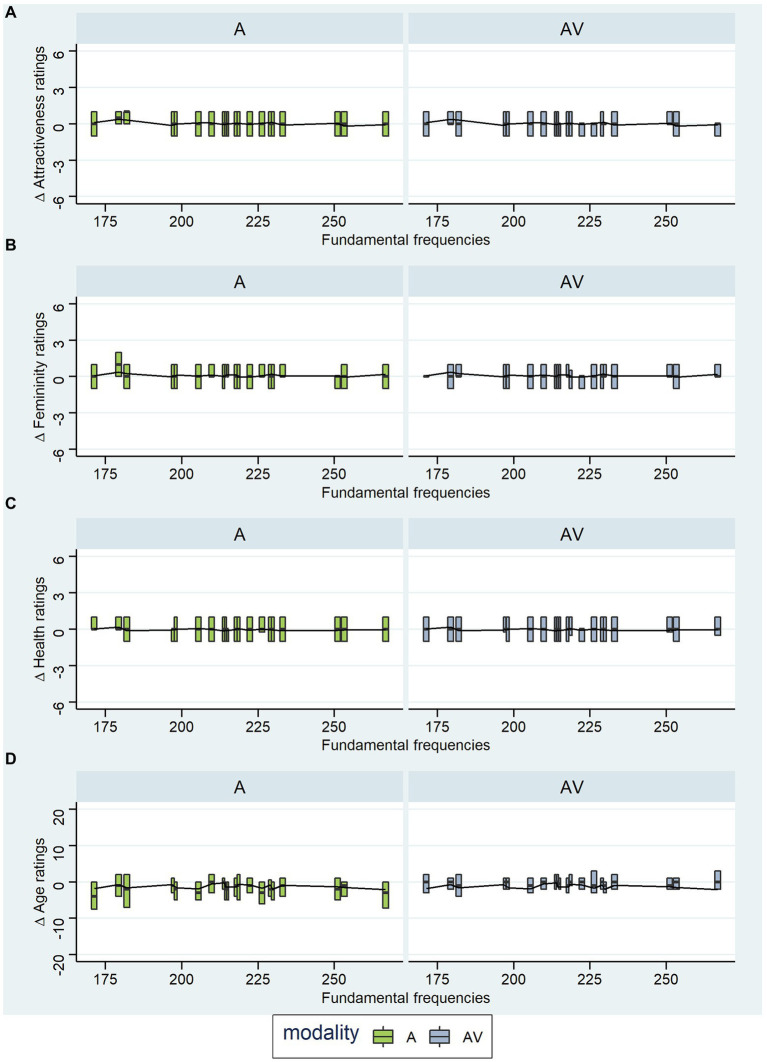
Exploratory Analyses: Rating Differences for Different Fundamental Frequencies. A and AV are the experimental blocks, whereby A = ratings of voices and AV = ratings of faces. Panel **(A)** shows rating differences for attractiveness ratings, **(B)** for femininity ratings, **(C)** for health ratings, and **(D)** for age ratings (in years). Whiskers and outliers were removed from the plots.

The same absolute change (e.g., 20 Hz) to different f_0_ values will not result in an equivalent absolute change in our perception of voice pitch, as pitch discrimination is relative with respect to f_0._ Therefore, a 20 Hz increment is perceived to be larger for a lower f_0_ and smaller for a higher f_0_. We wanted to investigate whether this perceptual difference is also echoed in the effect of pitch on rating responses. Therefore, we visualized the rating differences (for attractiveness, femininity, health, and age) grouped by the original fundamental frequencies that were present in the original voice recordings to reveal possible patterns (Figure 6). We would have expected a linear trend with f_0_ on the lower end of the range being more affected by the increment than f_0_ on the upper end of the range. However, all ratings show similar differences between original and increased voice pitch and no trend is detectable over different f_0_ values.

## Discussion

That judgments of attractiveness, femininity, health, and age rely on the sensory input of various sensory modalities, including the voice and the face, is undoubted. However, there is a lack of studies investigating the relative contribution and the cross-modal interaction of different sensory modalities on these social judgments when the perceiver is dealing with speaking faces and corresponding voices. The goal of the first part of this study was to disentangle the influence of voice pitch on voice judgments, namely attractiveness, femininity, health, and age by manipulating voice pitch and asking participants to rate unimodal auditory stimuli. We found that female voices with increased pitch were perceived as more feminine and younger compared to the original voice. Against our expectations, we did not find any effect of voice pitch on perceived attractiveness and health of voices. In the second part, we studied the cross-modality of the voice pitch effects, i.e., whether changes in the voice have a measurable influence on the perception of their speakers’ faces. Therefore, we investigated whether our voice pitch manipulation influences judgments of attractiveness, femininity, health, and age of the face in audiovisual stimuli. Consistent with the results from the first part of the study, we did also not find a cross-modal effect of voice pitch on the perceived attractiveness and health of faces. Voice pitch had a significant effect on the perceived femininity of voices presented alone, but this effect was not transferred to the perception of faces during audiovisual trials. Contrarily, increased voice pitch affected both perceived age of voices and perceived age of faces, suggesting a cross-modal effect of voice pitch on the perception of age.

### The Effect of Voice Pitch on Voice Judgments

Voice pitch is recognized as one of the main voice characteristics that distinguishes male and female voices, with higher pitch voices being perceived to be more feminine (in vowels; Feinberg et al., 2008). We replicated this finding suggesting that voice pitch remains a reliable parameter for femininity in non-vowel speech, with femininity ratings being on average significantly (but only slightly) higher in the increased pitch condition. Studies suggest that vocal breathiness (Van Borsel et al., 2007), vocal intensity (Dahl and Mahler, 2019), and vowel formant frequency (Gallena et al., 2017; Leung et al., 2021) also positively influence the perception of femininity in voices. It is possible that our findings for pitch could be due to an interaction with those voice characteristics as we allowed all aspects other than pitch to vary naturally between speakers. Future studies therefore might also consider other auditory parameters than just voice pitch when investigating voice perception and specifically vocal femininity.

Compared to other studies (e.g., Mook and Mitchel, 2019, where the total increase over 6 manipulation levels was 100 Hz), our f_0_ increase of 20 Hz was relatively small. While the pitch discrimination task revealed that participants could discriminate between original and increased voice pitch, one could still argue that the modest effect sizes we found for vocal femininity might be due to this rather small manipulation and that they could have been stronger if voice pitch was increased further, which would be interesting to consider in future studies.

Voice pitch not only allows us to distinguish between male and female voices, but it also represents an important feature to estimate a woman’s age. Female voice pitch significantly decreases with age in women, but it does not significantly change in men (Tykalova et al., 2021). In women, older women (mean age 75.2, range 60–89 years) and younger women (mean age 25.5, range 20–35) show differences in voice pitch of 15–25 Hz in several vowels (Torre and Barlow, 2009). While this is consistent with our finding that voices with increased pitch were judged to be younger than voices with original pitch, voices in our study were only judged to be an average of 2 years younger with a 20 Hz manipulation. It is also particularly interesting for the field, because previous studies (Mook and Mitchel, 2019) have used much larger manipulations, which seem to be far away from actual pitch differences within an individual over the lifespan. Considering the short-term voice pitch difference of around 5 Hz that has been found within individual women between non-fertile and fertile phases in the ovulatory cycle (Bryant and Haselton, 2009), 100 Hz increments seem even further from natural conditions. Our results, however, seem to emphasize a negative relationship between women’s voice pitch and perceived age of voices, while there have been mixed results on the relationship between voice pitch and perceived age, particularly in men, but also in women (for an overview see Torre and Barlow, 2009).

Surprisingly, we did not find an effect of increased voice pitch on perceived attractiveness and health of voices, which is contradictory to findings of prior studies: Vukovic et al. (2010a) found a negative correlation between f_0_ (values were wide-spread and ranged from around 170 HZ to 285 Hz) and a health risk index, suggesting that voice pitch is a cue to women’s long-term health. Collins and Missing (2003) found a positive correlation between voice pitch and ratings of vocal attractiveness, and Feinberg et al. (2008) found that participants prefer high pitch voices over average pitch voices. Mook and Mitchel (2019) also found a significant effect of voice pitch on attractiveness judgments of voices only when considering the difference over all their manipulation levels. However, not all levels seem to differ significantly in their study, e.g., voices with a f_0_ of 200 Hz and those with 220 Hz were rated as equally attractive (same mean); the largest difference in attractiveness judgments was between 160 and 180 Hz. In our study, f_0_ ranged from approximately 171 Hz to 267 Hz (Supplementary Table 1) in the original voices. However, most of our speakers (60%) had a f_0_ between 200 and 240 Hz (the range with the smallest differences in Mook and Mitchel, 2019), which suggests that maybe voice pitch does not have an effect on perceived attractiveness (and health) *per se*, but is rather restricted to specific frequency ranges. Moreover, most of the prior studies (Collins and Missing, 2003; Feinberg et al., 2008; Mook and Mitchel, 2019) used vowel stimuli to assess a pitch effect, and although vowels give sufficient information about harmonics and f_0_ (Patel et al., 2011), they differ from real-life speech utterances in their duration. Given that it seems to take around 1 s to form stable judgments of attractiveness from voices (see Zäske’s contribution in Krumpholz et al., 2021), the exposure time to a voice needed to create a stable rating is much longer than the duration of a vowel. Therefore, vowels might represent a first impression, but the voice judgment further unfolds until it becomes stable after 1 s. By using real-life speech utterances, our study presents results that might be closer to actual social situations. Of course, this also increases the risk that our results are influenced by factors beyond pure voice-based impressions such as accent, inflection, or emotional prosody (Rezlescu et al., 2015), which are however always present in natural stimuli.

### Cross-Modal Effects of Voice Pitch on Faces

Considering that increasing voice pitch had no unimodal effect on perceived attractiveness and health of voices, it is not surprising that we also failed to find cross-modal effects of voice pitch on perceived attractiveness and health of faces. Again, it would be interesting to investigate whether manipulations of other vocal parameters would have a cross-modal effect.

Mook and Mitchel (2019) suggested an asymmetrical integration, whereby vision is the dominant modality as facial attractiveness influences voice ratings, and audition is the non-dominant modality as voice attractiveness does not influence face perception. Similarly, in audiovisual integration of speech, the auditory stimulus seems to be altered by the visual component of the talking face (McGurk and MacDonald, 1976; Eskelund et al., 2011), and also in emotion expression recognition, the visual component seems to be dominant (Collignon et al., 2008). In the present study, perceived femininity of faces was not significantly affected by voice pitch, partly supporting prior findings that the visual domain is dominant in cross-modal interactions. Here, it is important to note that we did not investigate person perception (e.g., by asking how attractive this *person* is) as is typically done in speech or emotion expression research, but face perception (by asking how attractive this *face* is). Therefore, we cannot draw conclusions about actual audiovisual integration, but only speculate. Future studies should address person perception to see if these findings on cross-modal interactions can also be detected in audiovisual integration.

Nevertheless, the effect could also be explained alternatively. Femininity ratings in both blocks, audio and audiovisual, showed a similar pattern (see Figure 4 for the audio block and Figure 5 for the audiovisual block), but did not reach significance in the ratings of faces. So, one could argue that there would be a cross-modal effect, but it unfolds only with a stronger manipulation than 20 Hz. In fact, given that relative differences in frequency are relevant for auditory perception, it is better to consider relative differences in frequency (measured in Cents) instead of absolute differences in Hz. For example, an increase from 171 to 191 Hz (the lowest f0 occurring in our dataset) means an increase in relative terms of 191 Cents, which is just below two semitones, while an increase from 267 to 287 Hz (our highest f0) means much less in relative terms, a little more than a semitone. For the auditory system, pitch differences larger than a minor third (equivalent to 300 Cents) become clearly distinguishable, since from here on a critical band width is exceeded (Fastl and Zwicker, 2007). However, one should ask how meaningful this pitch “supersizing” is in relation to the natural changes mentioned above that occur in an individual woman’s voice pitch over her lifetime or short-term changes during the menstrual cycle (Bryant and Haselton, 2009; Torre and Barlow, 2009).

Studies showed that people are generally pretty accurate when they have to estimate age from a face or a voice into an age range (for a review see Moyse, 2014). However, Amilon et al. (2007) found that people were more accurate at estimating the age based on face information from still images and from videos with no audio track than they are when given only voice information in the form of 5 sec speech recordings. They also included a video condition with simultaneous information of faces and voices and found that voice information did not improve age estimations. Surprisingly, in the present study, voice pitch did affect age estimations of faces, with women’s faces being perceived as younger when voice pitch was increased. This suggests that the voice might play a more important role in age estimation than previously assumed. It remains unclear whether similar results can be found when raters were asked to estimate the age of the person and not just their voice or face, which would give more insight into audiovisual integration and the relative contribution of each sensory modality, respectively.

### Future Directions

Future studies should investigate how voice pitch relates to the judgment of the whole stimulus, namely the person, instead of the respective sensory modalities, and whether auditory and visual information are integrated in this judgment. While such an investigation is beyond the scope of this paper, some speculation is possible. One possibility is that the voice contributes to some but not all person judgments, but it might not be strong enough to overwrite the contribution of the face, suggesting that the face is mostly, but maybe not always the dominant sensory modality.

Again, we let other voice parameters such as formant dispersion or breathiness vary naturally to keep our stimuli as natural as possible. However, it may well be the case that changing f_0_ does not affect all voices equally strongly due to inter-individual variation in other voice parameters. Considering our results, voice pitch might not be the most important voice parameter, specifically regarding perceived attractiveness and health. Future studies should target other voice parameters than voice pitch and investigate their influence on face, voice, and person perception. While our voice pitch manipulation was perceptually discriminable (Figure 2) and, considering natural changes in voice pitch, reasonable, it would also be interesting for further studies to repeat similar experiments with bigger increments in voice pitch.

Last, we want to note that attractiveness judgments are based on more than faces and voices. Odor cues (Havlicek et al., 2008; Lobmaier et al., 2018), visual perception of the body (Kościński, 2013), gaze direction (Ho et al., 2018; Kaisler et al., 2020), and emotional expression (Lindeberg et al., 2019; Kaisler et al., 2020) for example also play an important role and should be addressed in future studies.

## Data Availability Statement

The datasets presented in this study can be found in online repositories. The names of the repository/repositories and accession number(s) can be found at: https://doi.org//10.17605/OSF.IO/DUTPR.

## Ethics Statement

The studies involving human participants were reviewed and approved by Ethics Committee of the University of Vienna. The patients/participants provided their written informed consent to participate in this study.

## Author Contributions

CK, CQ, KA, LF, and HL: conceptualization. CK, CQ, KA, and HL: methodology. CK, KA, and CR: resources. CK and CQ: formal analysis and visualization. CK: writing – original draft preparation. CK, CQ, CR, LF, and HL: writing – review and editing. All authors contributed to the article and approved the submitted version.

## Funding

This work was supported by the Vienna Science and Technology Fund (WWTF) CS18-021 (principal investigator: LF).

## Conflict of Interest

The authors declare that the research was conducted in the absence of any commercial or financial relationships that could be construed as a potential conflict of interest.

## Publisher’s Note

All claims expressed in this article are solely those of the authors and do not necessarily represent those of their affiliated organizations, or those of the publisher, the editors and the reviewers. Any product that may be evaluated in this article, or claim that may be made by its manufacturer, is not guaranteed or endorsed by the publisher.

## Footnotes

^1^ https://osf.io/dutpr/?view_only=0e5b17f84bae4a25aa91432ae22422e2
