# Supplementary material for: The Effects of Pitch Manipulation on Male Ratings of Female Speakers and Their Voices
Source: Front Psychol. 2022 Jul 7;13:911854. doi: 10.3389/fpsyg.2022.911854 (PMC9302589; doi:10.3389/fpsyg.2022.911854)
Supplement: Supplementary file 1 [file Data_Sheet_1.PDF]

Supplementary Material

Table 1

*Overview of fundamental frequency (**f_0**), minimum pitch, maximum pitch, and pitch range for all stimuli.*

Stimulus	f_0	Minimum pitch	Maximum pitch	Pitch range
006_M	171.36	120.29	237.12	116.82
035_M	179.37	146.42	221.19	74.78
012_M	182.02	131.34	236.25	104.92
014_M	196.70	102.15	262.37	160.21
026_M	198.46	164.64	572.99	408.36
013_M	205.47	156.60	243.92	87.32
023_M	209.81	170.89	261.06	90.17
031_M	214.12	108.62	262.43	153.81
024_M	214.32	190.17	251.20	61.04
004_M	214.63	103.50	258.98	155.49
011_M	218.04	162.27	352.43	190.16
027_M	218.29	109.73	346.41	236.68
032_M	222.29	102.57	578.15	475.58
009_M	226.31	160.03	527.97	367.94
010_M	228.98	145.31	480.75	335.45
015_M	229.79	188.52	579.71	391.19
005_M	233.03	170.76	598.56	427.80
034_M	251.08	100.87	438.22	337.35
019_M	253.24	153.94	597.20	443.27
020_M	266.78	145.14	356.23	211.09

Note: F_0 is calculated as the mean of f_0 from one of the voice recordings, the minimum and the maximum pitch are the minimum/maximum values from one of the voice recordings. The pitch range is calculated as the difference between minimum and maximum voice pitch. Stimulus names are aligned/matched with the ViTaFa stimulus names in order to assign them.

Table 2

Overview of the applied models for all ratings in the both blocks. Included models are shown in bold.

a) Audio block

Rating	Model term	Final null model	Exclusion criterium
Attractiveness	lmer(rating ~condition + (1+condition|subject_nr) + (1+condition|stimulus_nr))	lmer(rating ~(1+condition|subject_nr) + (1+condition|stimulus_nr))	included
Femininity	lmer(rating ~condition + (1+condition|subject_nr) + (1+condition|stimulus_nr))		Failed to converge (stimulus_nr shows correlation of 1)
	**lmer(rating ~condition + (1+condition|subject_nr)**	lmer(rating ~(1+condition|subject_nr)	included
Health	lmer(rating ~condition + (1+condition|subject_nr) + (1+condition|stimulus_nr))		Boundary fit (overfitting; stimulus_nr shows correlation of 1)
	**lmer(rating ~condition + (1+condition|subject_nr)**	lmer(rating ~(1+condition|subject_nr)	included
Age	**lmer(rating ~condition + (1+condition|subject_nr) + (1+condition|stimulus_nr))**	lmer(rating ~(1+condition|subject_nr) + (1+condition|stimulus_nr))	included

b) Video block

Rating	Model term	Final null model	Exclusion criterium
Attractiveness	lmer(rating ~condition + (1+condition|subject_nr) + (1+condition|stimulus_nr))		Boundary fit (overfitting; stimulus_nr shows correlation of 1)

	**lmer(rating ~condition + (1+condition|subject_nr)**	lmer(rating ~(1+condition|subject_nr)	included
Femininity	lmer(rating ~condition + (1+condition|subject_nr) + (1+condition|stimulus_nr))		Boundary fit (overfitting; stimulus_nr shows correlation of 1)
	**lmer(rating ~condition + (1+condition|subject_nr)**	lmer(rating ~(1+condition|subject_nr)	included
Health	lmer(rating ~condition + (1+condition|subject_nr) + (1+condition|stimulus_nr))		Boundary fit (overfitting; stimulus_nr shows correlation of 1)
	**lmer(rating ~condition + (1+condition|subject_nr)**	lmer(rating ~(1+condition|subject_nr)	included
Age	lmer(rating ~condition + (1+condition|subject_nr) + (1+condition|stimulus_nr))		Boundary fit (overfitting; stimulus_nr shows correlation of 1)
	lmer(rating ~condition + (1+condition|subject_nr)		Boundary fit (overfitting; stimulus_nr shows correlation of 1)
	**lmer(rating ~condition + (1|subject_nr)**	lmer(rating ~(1|subject_nr)	included
